# Personalized Development of Antisense Oligonucleotides for Exon Skipping Restores Type XVII Collagen Expression in Junctional Epidermolysis Bullosa

**DOI:** 10.3390/ijms22073326

**Published:** 2021-03-24

**Authors:** Michael Ablinger, Thomas Lettner, Nicole Friedl, Hannah Potocki, Theresa Palmetzhofer, Ulrich Koller, Julia Illmer, Bernadette Liemberger, Stefan Hainzl, Alfred Klausegger, Manuela Reisenberger, Jo Lambert, Mireille Van Gele, Eline Desmet, Els Van Maelsaeke, Monika Wimmer, Roland Zauner, Johann W. Bauer, Verena Wally

**Affiliations:** 1 Research Program for Molecular Therapy of Genodermatoses, EB House Austria, Department of Dermatology and Allergology, University Hospital of the Paracelsus Medical University, 5020 Salzburg, Austria; m.ablinger@salk.at (M.A.); thomas.lettner@gmail.com (T.L.); nicole_friedl@gmx.at (N.F.); potockiha@gmx.de (H.P.); theresa.palmetzhofer@gmx.at (T.P.); u.koller@salk.at (U.K.); ju.illmer@crcs.at (J.I.); b.liemberger@crcs.at (B.L.); s.hainzl@salk.at (S.H.); a.klausegger@salk.at (A.K.); m.reisenberger@salk.at (M.R.); mo.wimmer@crcs.at (M.W.); rolan.zauner@salk.at (R.Z.); joh.bauer@salk.at (J.W.B.); 2 Department of Dermatology and Allergology, University Hospital of the Paracelsus Medical University, 5020 Salzburg, Austria; 3 Department of Dermatology, Ghent University Hospital, 9000 Ghent, Belgium; jo.lambert@uzgent.be (J.L.); mireillevangele@gmail.com (M.V.G.); eline.desmet@d-n.be (E.D.); Els.VanMaelsaeke@ugent.be (E.V.M.)

**Keywords:** molecular therapy, junctional epidermolysis bullosa, type XVII collagen, splice mutation, antisense oligonucleotides, exon skipping, topical therapy, liposomes

## Abstract

Intermediate junctional epidermolysis bullosa caused by mutations in the *COL17A1* gene is characterized by the frequent development of blisters and erosions on the skin and mucous membranes. The rarity of the disease and the heterogeneity of the underlying mutations renders therapy developments challenging. However, the high number of short in-frame exons facilitates the use of antisense oligonucleotides (AON) to restore collagen 17 (C17) expression by inducing exon skipping. In a personalized approach, we designed and tested three AONs in combination with a cationic liposomal carrier for their ability to induce skipping of *COL17A1* exon 7 in 2D culture and in 3D skin equivalents. We show that AON-induced exon skipping excludes the targeted exon from pre-mRNA processing, which restores the reading frame, leading to the expression of a slightly truncated protein. Furthermore, the expression and correct deposition of C17 at the dermal–epidermal junction indicates its functionality. Thus, we assume AON-mediated exon skipping to be a promising tool for the treatment of junctional epidermolysis bullosa, particularly applicable in a personalized manner for rare genotypes.

## 1. Introduction

Epidermolysis bullosa (EB) represents a group of genetic disorders with severe skin fragility. It is phenotypically characterized by blisters and erosions of the skin and mucous membranes, resulting from mechanical trauma that leads to tissue separation at the dermo–epidermal junction (DEJ) [1]. Depending on the localization of the tissue disruption within the DEJ, four major subtypes can be phenotypically distinguished: EB simplex (EBS), junctional EB (JEB), dystrophic EB (DEB) and Kindler EB (KEB) [1]. The different subtypes are associated with a broad spectrum of extracutaneous manifestations [2,3,4], and the severe subtypes go along with a reduced life expectancy [5]. The degree or magnitude of the phenotypic manifestations (severe vs. intermediate forms) depends on the genes involved, the type of mutation and, consequently, the residual protein expression, the mode of inheritance (dominant vs. recessive) and the affected site of the body (generalized vs. localized forms). Common to all subtypes, however, is the severe negative impact on the patients’ life quality (QoL).

At present, no cure is available for any subtype of EB, but several clinical trials are currently being conducted [6,7,8]. The majority of therapeutic developments are not of causal but of palliative nature, aiming to reduce blister formations [9], to improve wound healing [10] and to predict or delay carcinogenesis [11]. However, only gene therapeutic approaches will have the potential to reverse the disease phenotype causally and permanently [12]. Despite the high efforts that are made towards the development of gene therapies for different EB subtypes, first marketing approvals are still being awaited and are not expected for all EB types in the near future. This renders antisense oligonucleotide (AON)-induced exon skipping an important alternative, as AON-based treatments have already been approved for distinct genetic diseases like spinal muscular atrophy [13,14].

The genetic basis of JEB are mutations in either one of the laminin genes (*LAMA3*, *LAMB3* and *LAMC2*); the integrin genes (*ITGA3*, *ITGA6* and *ITGB4*) or collagen 17 (*COL17A1, syn. BPAG2* and *syn. BP180*) [1], all of which are components of hemidesmosomes, a multiprotein transmembrane complex that connects basal keratinocytes intracellularly to keratin filaments and extracellularly to the underlying basement membrane zone (BMZ) [15]. In the case of the structural impairment of hemidesmosomes (HD), skin separation occurs within the lamina lucida of the DEJ, which is a characteristic of JEB. JEB patients require lifelong medical attention. A therapeutic intervention to improve intermediate JEB patients’ QoL is therefore urgently needed.

In this study, we present the successful correction of a *COL17A1* splice site mutation (c.380-1G>A) during mRNA maturation using an AON-mediated approach. The observation that the minor residual expression of collagen 17 (C17) is associated with mild phenotypes is a promising indicator for the feasibility of AON therapies, even if locally applied in a liposome-based formulation [16,17]. We show that this method effectively skips the affected exon and excludes it from pre-mRNA processing, thereby restoring the wild-type open reading frame (ORF), which leads to the expression of a slightly shorter but functional C17 protein. Furthermore, we employed a liposome-based protocol for the topical delivery of antisense molecules into human keratinocytes and 3D skin equivalents. This work therefore represents an important step in the development of a treatment for patients suffering from rare variants of JEB.

## 2. Results

### 2.1. A Homozygous COL17A1 Nonsense Mutation Leads to Loss of C17 Expression

An eight-year-old patient presented to our clinic with blisters and erosions typical for the intermediate JEB subtype. A mutational analysis revealed a homozygous G > A transition at the *COL17A1* intron 6/exon 7 acceptor splice site (c.380-1G>A). Due to this mutation, the splicing machinery utilizes the next available AG dinucleotide, which is located 16 nucleotides downstream within the exon 7 pre-mRNA sequence, as the new acceptor site for intron excision and exon ligation, leading to the deletion of the first 16 nucleotides (∆16) of exon 7. This deletion results in a frameshift, and subsequently, the aberrantly spliced mRNA runs into a premature termination codon (PTC) that triggers nonsense-mediated decay (NMD) (Supplementary Materials Figure S1A,B). Consequently, C17 expression levels are significantly reduced at the mRNA and the protein level (Figure S1C,D). Considering the given genetic background, we assumed that the induction of exon 7 skipping (36 nts) using AONs would restore the ORF, resulting in the re-expression of a functional, although slightly shortened, C17 protein.

### 2.2. Design of AONs Capable of Exon 7 Skipping in Combination with A Liposomal Delivery System

**Figure 1:**
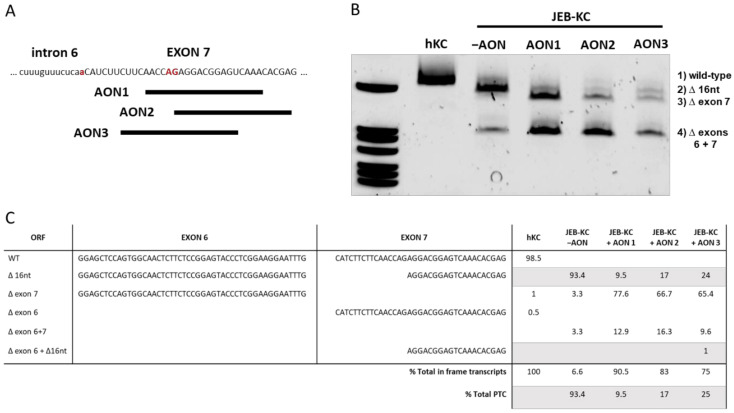
Antisense oligonucleotides (AON)-induced exon skipping. (**A**) Three AONs were designed to bind complementary to the cryptic exon 7 splice acceptor site (red upper case letters). The mutation at the wild-type splice junction isindicated as red lower case letter. (**B**) Exon skipping patterns observed in AON-treated vs. untreated (−AON) junctional epidermolysis bullosa patient-derived keratinocyte line (JEB-KC) compared to healthy control KC (hKC). Untreated JEB-KC show a distinct band that corresponds to the deletion of 16 nts of exon 7 (∆16 nt). All AON-treated samples show splicing products corresponding to the skipping of exon 7 (∆7) or exon 6 and exon 7 in combination (∆6+7). The latter was also found in untreated JEB-KC. All bands were analyzed by Sanger sequencing. (**C**) Next-generation sequencing (NGS) results of *COL17A1* mRNA splice variants. All numbers are given as percentages, and grey lines indicate that transcripts have an open reading frame (ORF) divergent from the wild type.

Considering the first promising reports of antisense-mediated exon skipping at the time, for instance, as a treatment option for diseases like muscular dystrophy [18,19], we used online bioinformatic tools (Rescue ESE) to design three different antisense oligonucleotides (AONs). All three AONs bind complementary to the endogenous exon 7 splice acceptor site in *COL17A1* pre-mRNA (Figure 1A), which also contains a cluster of exonic splice enhancer sequence motifs (Figure S2) [20]. We hypothesized that masking the aberrant binding site for the splice apparatus will potentially promote skipping of the 36-nts-long exon 7 and restoration of the reading frame.

In addition, as almost any therapeutic approach in the EB field and in dermatology in general has to face the challenging task of the efficient topical delivery of drugs through the outer barrier of the epidermis, we aimed to use a delivery method that comes with low toxicity, as well as properties well-suited for skin transfection. Therefore, we investigated the applicability of cationic DDC642 liposomes in delivering AONs in 2D and 3D experiments, since they have been shown before to efficiently deliver short interfering (si)RNAs into hyperkeratotic psoriatic skin [21,22].

Initially, we tested whether DDC642 liposomes could be successfully complexed with different AONs in a gel retardation assay. This experiment revealed a high capacity of AONs and liposomes to form lipoplexes (Figure S3A) and the high capacity of lipoplexes to deliver AONs to keratinocytes (KCs), going along with the low cytotoxicity (Figure S3B). Of note, the transfection of JEB-KC with Cy5-labeled AONs resulted in approximately 90% Cy5-positive cells in fluorescence microscopy (Figure S3C). Further, AONs clearly accumulated in the nucleus, which is a prerequisite for modulating the splicing process. In addition, we showed that our Cy5-AON lipoplexes were able to penetrate human skin, where Cy5-AONs reached the C17-expressing basal cell layer and colocalized to the keratinocyte nuclei in all skin sections (Figures S4 and S5). Even though a higher level of penetration was achieved using tape-stripped skin, the delivery was also successful into intact skin that had not been pretreated. Of note, as the target skin layer is the epidermis, a delivery of the AONs exclusively to keratinocytes, as observed in non-pretreated skin, is preferable, as this would also reduce the risk of systemic distribution.

Investigation of the ability of the AONs to induce exon skipping in a JEB patient-derived keratinocyte line (JEB-KC) confirmed the efficient exclusion of exon 7 during mRNA maturation (Figure 1B). Unexpectedly, we also detected mRNA transcripts with the concomitant exclusion of exons 6 and 7, which was confirmed by Sanger sequencing. Interestingly, this also occurred in untreated patient keratinocytes at a very low level, which was confirmed by next-generation sequencing (NGS). There, 3.3% of reads revealed the concomitant skipping of exons 6 and 7 in untreated JEB-KCs. In addition, skipping of the 48-nts-long exon 6 or exon 7, respectively, was found in 1.5% of reads from healthy control KCs (hKC). This observation points toward the naturally occurring skipping of single exons in the intracellular domain of C17, supporting the concept of our approach to generate a functional C17 protein by the skipping of exon 7. Overall, the treatment of JEB-KCs with any of the three AONs resulted in a shift of C17 transcripts with the wild-type reading frame from 6.6% (untreated JEB-KCs) to up to 90.5% (Figure 1C).

### 2.3. AON-Induced Exon Skipping Restores Type XVII Collagen Expression

Crucial to antisense-induced exon skipping is the maintenance of the functionality of the truncated protein, for which we considered the overall expression levels, correct transmembrane integration and shedding of the ectodomain as hallmarks.

**Figure 2:**
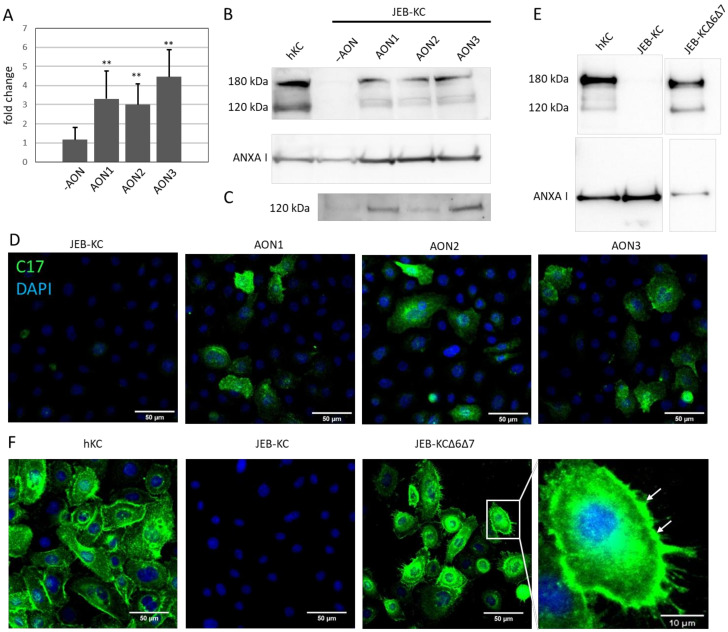
AON-mediated type XVII collagen expression. (**A**) Semiquantitative (Sq)RT-PCR showed the restoration of *COL17A1* expression in JEB-KC upon AON treatment. *GAPDH* was used as the reference. Fold change over untreated JEB-KC (-AON) was calculated using the 2^−∆∆Ct^ method [23]. Unpaired, two-sided Student’s *t*-test was performed (*n* = 6), and *p* ≤ 0.05 was considered significant (** *p* < 0.01). (**B**) Western blot analysis of whole-cell lysates. Wild-type hKC showed two distinct type XVII collagen bands at 180 and 120 kDa that are absent in JEB-KC and reappear upon AON treatment. Annexin I (ANXAI) was used as the loading control. (**C**) The 120-kDa band reappeared in a conditioned culture medium of AON-treated JEB-KC. (**D**) In immunofluorescence microscopy, the images show that the expression of type XVII collagen is restored upon AON treatment. Scale bars: 50 µm. (**E**) Western blot analysis showed the expression of both the 120-kDa and the 180-kDa collagen 17 (C17) variants from the C17 lacking exons 6 and 7 (Δ6Δ7) expression cassette. (**F**) In addition, JEB∆6∆7 stained positive for type XVII collagen and showed C17 integration into the cell membrane (magnified section, white arrows). Scale bars: 50 µm, magnification: 10 µm.

In JEB-KC, the AON-induced skipping of exon 7 (∆ exon 7) or exons 6 and 7 (∆ exons 6+7) in combination led to significantly increased levels of C17 mRNA (*p* < 0.01), as shown by semiquantitative (sq)RT-PCR (Figure 2A). Furthermore, in Western blotting experiments, the full-length 180-kDa band and the 120-kDa ectodomain band reappeared in the analysis of the whole cell lysates after the AON treatment (Figure 2B), and the 120-kDa ectodomain became detectable in the cell culture supernatants (Figure 2C). In addition, the expression of C17 was confirmed by immunofluorescence microscopy (Figure 2D).

In order to further investigate if the loss of exons 6 and 7 had functional consequences, we stably transduced JEB-KCs with a *COL17A1* expression cassette lacking exons 6 and 7 (JEB∆6∆7). We investigated whether the loss of both exons interfered with the expression of a functional protein and observed expression of C17 by Western blot analysis and correct integration into the cell membrane in immunofluorescence microscopy (Figure 2E,F). These results indicate the functionality of the truncated protein.

### 2.4. Treatment of 3D Skin Equivalents with AONs Results in A Stable Epidermis and Correct Localization of Type XVII Collagen

**Figure 3:**
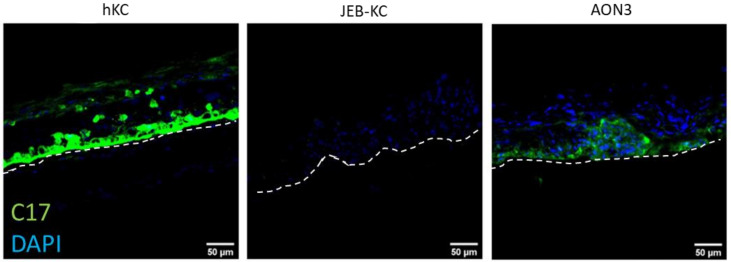
Generation of 3D skin equivalents from AON-treated JEB-KC. Skin equivalents were generated from hKC and JEB-KC. Upon treatment with AON3, the deposition of C17 became detectable within the basal layer of the epidermis. Scale bar: 50 µm.

In order to investigate whether the AONs are able to restore C17 expression in a stratified epidermis, we generated 3D skin equivalents (SE) using JEB-KCs that were treated with AON lipoplexes before seeding onto a dermal matrix, as well as before lifting the SE to the air–liquid interface. The immunostaining of cryosections showed the expression of C17 in AON-treated SEs and their predominant localization to the basal layer of the epidermis. In contrast, untreated SEs showed no C17 expression (Figure 3). These results indicate that C17 re-expression can be achieved by also treating JEB-KCs in a 3D skin model, which also substantiates the applicability of this approach in a clinical setting.

## 3. Discussion

In this project, we investigated the feasibility of AONs to restore C17 expression through the skipping of *COL17A1* exon 7. Our experiments showed that the deleterious effect of the c.380-1G>A splice mutation can be mitigated by AON treatment, resulting in the expression of a slightly shorter but functional protein. Immunofluorescence images revealed that the truncated protein integrated into the cell membrane of JEB-KC in 2D and was predominantly expressed in the basal layer of 3D skin equivalents. The observation that exons 6 and 7 are also naturally skipped in healthy keratinocytes supported our hypothesis that these exons may be dispensable.

Type XVII collagen is an essential component of HDs, which are not just anchoring basal keratinocytes to the BMZ; the intracellular part of HDs is of crucial importance for the organization of the keratin cytoskeleton of epithelial cells [15]. For the correct assembly of the cytoplasmic parts of HDs, the binding of type XVII collagen to the bullous pemphigoid antigen-230 and plectin is required. Contact to its interaction partners is established via binding to the Y-domain, a homologous domain found in BP230 and plectin. During HD assembly, the amino terminal part of type XVII collagen binds to BP230 and plectin with a stretch of 280 amino acids, covering residues 180 to 460 [24]. Of note, the 28 amino acids encoded by exons 6 and 7 are located at residues 110 to 137, and their deletion is therefore unlikely to affect the binding to BP230 and plectin or the intracellular assembly of HDs and the correct organization of the cytoskeleton. This assumption is corroborated by a case report on a patient with mutations leading to a deletion of the intracellular part of type XVII collagen from exon 3 to exon 15, which resulted in a mild clinical phenotype that resembled the simplex type of EB rather than JEB [25]. In addition, Kirtisi et al. showed that a residual amount of 12–14% of functional type XVII collagen is sufficient to generate a mild JEB phenotype, and Ruzzi et al. observed that C17 levels as low as 3% to 4% of normal were associated with only minor blistering [17,26].

Furthermore, Paasmoij et al. reported on a JEB patient in whom the natural skipping of *COL17A1* exon 30 compensated a nonsense mutation within this exon, also resulting in a mild phenotype [27].

AON-induced exon skipping was shown to be a promising tool for the correction of genetic defects at the mRNA level for deleterious diseases like Duchenne’s muscular dystrophy and spinal muscular atrophy [13,28]. The methodical approach also offers options for therapeutic intervention in a variety of other diseases, such as cardiomyopathy [29], Pompe disease [30], Stargardt disease [31] or p53 pathway-related diseases [32], just to name a few. The technology has already entered several clinical trials, and a growing list of antisense-based therapeutics are already FDA-approved (e.g., Spinraza^®^ [14]. Regarding EB, AON-induced exon skipping has successfully been shown for frequently mutated exons 73, 80 and 105 of *COL7A1*, and it is one out of various therapeutic approaches that are currently in development [6,7,33,34,35]. The fortunate fact that most of *COL17A1* and *COL7A1* exons are in frame and that several of them are dispensable for normal or sufficient protein function [36,37] has fueled the development of AON and exon skipping approaches primarily for the JEB and DEB subtypes of EB [35,36,38,39]. Considering RDEB, a clinical trial is currently ongoing using the exon 73 targeting AON QR-313 that will be applied onto EB wounds (NCT03605069) [33]. The administration of AONs directly onto wounds is the simplest and probably safest way of delivery, which was therefore one of the main premises of this study. The rare development of extracutaneous complications in intermediate JEB patients indicates the feasibility of using a liposome-based topical route of administration for QoL improvements.

Liposomes are organized structures formed through the self-assembly of amphiphilic phospholipids in an aqueous environment, with a morphology that resembles cellular membranes. Liposomes have been used in cosmetics for decades, and their potential to be used for drug delivery has been shown in various studies. However, liposomes also come with distinct limitations, like a restricted stability, limited capacity of drug uptake and the risk of side effects as a result of liposome accumulation [40]. For topical delivery, we used cationic liposomes, as they can efficiently interact with negatively charged keratinocyte membranes, potentially facilitating internalization, which we and others could successfully show for DDC642 liposomes [22]. Of note, the feasibility of complexing AONs with liposomes and the delivery of the AONs to epidermal keratinocytes even in the presence of an intact stratum corneum indicates that both the application of our lipoplexes onto wounds or onto intact skin might be feasible treatment strategies. The presented approach may thus pose a versatile, easy to design and relatively cheap way to provide this therapy to patients, even if they have an ultra-rare genotype that requires a personalized approach.

Thus, antisense-induced exon skipping approach could bridge the gap until a permanent cure can be provided for EB patients.

## 4. Materials and Methods

### 4.1. Cell Lines and Cell Culture

Primary keratinocytes derived from skin biopsies were immortalized by transduction with HPV16 E6/E7, as described elsewhere [41]. Immortalized cell lines were cultivated in serum-free medium (CELLnTEC, #CnT-PR, Bern, Switzerland) at 37 °C and 5% CO_2_ in a humidified atmosphere. Immortalized keratinocytes, derived from a healthy donor (hKC), were used as the control.

### 4.2. Generation of A JEB∆6∆7 Cell Line

A *COL17A1∆6∆7* expression cassette was cloned into the retroviral pMXs-IRES-Blasticidin vector (Cell Biolabs, #RTV-016, San Diego, CA, USA) using the Gibson assembly cloning kit (NEB, #E5510S, Frankfurt am Main, Germany). Full-length *COL17A1* cDNA was amplified from healthy keratinocytes and used as the template. The following primer pairs were used:

F1_ex6+7_fw: 5′-caggcctcgagggccggcgcgccgcATGGATGTAACCAAGAAAAACAAACG-3′
F1_ex6+7_rv: 5′-cgaatttcactctCTTCATACGCATGGCGGG-3′
F2_fwd: 5′-cggaaggaatttgAGAGTGAAATTCGAGTTCGAC-3′
F2_rev: 5′-aatgccaagagccCCTGGAACACCTGGATCAC-3′
F3_fwd: 5′-caggtgttccaggGGCTCTTGGCATTCCTAG-3′
F3_rev: 5′-ggaggggggggggcggaatttacgtagcTCACGGCTTGACAGCAATAC-3′

JEB-KC were retrovirally transduced, as recently described [41]. Genomic integrations were verified by Sanger sequencing.

### 4.3. AON Design

For the selection of exonic splice enhancer (ESE) sequences, the *COL17A1* intron 6/exon 7 nucleotide sequence was submitted to ESEfinder 3.0 (http://krainer01.cshl.edu/cgi-bin/tools/ESE3/esefinder.cgi?process=home, accessed on 1 December 2016) and to the Rescue ESE online tool (http://hollywood.mit.edu/burgelab/rescue-ese/, accessed on 1 December 2016). Three individual AONs of 20 nts in length were predicted and designed to bind complementarily to the exon 7 cryptic acceptor splice site, covering different stretches of the identified ESE motif. The AONs contain 2′-O-methyl, as well as phosphorothioate modifications, and for each AON, a 5′-Cy5 modified version was provided for fluorescence microscopy. All AONs were purchased from Microsynth AG, Balgach, Switzerland (http://www.microsynth.ch, accessed on 1 December 2016).

AON1_C17_20: 5′-TTTGACTCCGTCCTCTGGTT-3′
AON1_C17_20_Cy5: 5′-Cy5-TTTGACTCCGTCCTCTGGTT-3′
AON2_C17_20: 5′-TCGTGTTTGACTCCGTCCTC-3′
AON2_C17_20_Cy5: 5′-Cy5-TCGTGTTTGACTCCGTCCTC-3′
AON3_C17_20: 5′-CTCCGTCCTCTGGTTGAAGA-3′
AON3_C17_20_Cy5: 5′-Cy5-CTCCGTCCTCTGGTTGAAGA-3′

### 4.4. Splice Pattern Evaluation

In order to evaluate the splice pattern of AON-treated and untreated JEB-KC, as well as hKC, we transfected JEB-KC with AON1, AON2 and AON3 in individual experiments. Forty-eight hours after transfection, RNA was isolated using the RNeasy Mini Kit (QIAGEN, #74104, Hilden, Germany). cDNA was prepared from 1-µg total RNA using the iScript™ cDNA Synthesis Kit (Bio-Rad, #1708891, Hercules, CA, USA). RT-PCR was performed using a primer pair amplifying *COL17A1* cDNA from exon 5 to exon 9. PCR products were analyzed on a 6% TBE gel (Invitrogen, #EC6265BOX, Carlsbad, CA, USA). Forward primer: C17_E5_fw_CTGGAAGCACACGAGGCCATGC and reverse primer: C17_E9_rv_GTGGGGCTCACACTTGCCGAT.

### 4.5. DDC642 Liposome Production

DDC642 liposomes were generated as described by Desmet et al. [21]. Briefly, DOTAP (Sigma, #D6182-50mg, St.Louis, CA, USA), DOPE (Sigma, #42490-2.5 mL) and cholesterol (Sigma, #C8667-500 mg) were combined in a 6:4:2 ratio, incubated at 37 °C and resuspended in an EtOH/HEPES buffer. After incubation for 24 h at room temperature, the lipid solution was extruded 30 times through a 100-nm pore-sized polycarbonate membrane (Sigma, #Z373419-50EA) using a LiposoFast Liposome Factory (Sigma, #Z373400).

### 4.6. Gel Retardation Assay

The best conditions for lipoplex (DDC642 liposomes + nucleic acid) formation, which is dependent on the chemical properties of the oligonucleotide (e.g., length, net charge and chemical modifications), were determined using a gel retardation assay. For that, a serial dilution (0.5 µg, 1 µg, 2 µg and 4 µg) of all three AONs was prepared in a final volume of 6-µl ddH_2_O. Each dilution was combined with 4-µl DDC642 (4 µg/µL) under gentle vortexing for 15 s and incubation at room temperature for 10 min. GelRed^®^ Nucleic Acid Gel Stain (Biotium, Fremont, CA, USA) was used to visualize the oligonucleotides; the mixtures were electrophoretically analyzed on a 1% agarose gel and visualized under UV light.

### 4.7. Cell Viability

The day before the experiment, 1.5 × 10^4^ JEB-KC per well were seeded into a 96-well tissue culture plate with 4 replicates for the untreated and liposome-treated cells. After 24 h, different amounts of empty liposomes were added. Viability was determined by incubating cells with 25 µL of 5-mg/mL MTT substrate (Abcam, #ab146345, Cambridge, UK) and 100-µL serum-free medium for 1 to 2 h. Medium was aspirated, and 100-µL lysis solution were added (6:1 DMSO/0.1-M glycine/NaOH, pH10). The plate was incubated for 10 min on a plate shaker with 500 rpm. Absorbance was measured at 492 nm. Data were normalized by subtraction of the background reference signal from all values. Viability in percent was calculated as treated/untreated ×100. Unpaired, two-sided Student’s *t*-test was used to evaluate the statistical significance.

### 4.8. Immunofluorescence Microscopy

hKC, JEB-KC and JEB∆6∆7 cells (1.5 × 10^5^) were seeded per well into 2-well chamber slides (Nalge Nunc International, Lab-Tek^®^ II Chamber Slide™ system, #154461, Rochester, NY, USA) and cultivated for 48 h in serum-free medium (CELLnTEC, #CnT-PR). At confluence, cells were fixed with ice-cold methanol for 10 min, washed with PBS and blocked with 5% BSA (Sigma, #A3294) in PBS for 1 h. The primary antibody against type XVII collagen (abcam, #ab184996) was incubated overnight at 4 °C. After washing with PBS, cells were incubated with Alexa Fluor 488-conjugated secondary antibody (Thermo Fisher Scientific, #A11008, Waltham, MA, USA) and DAPI (Sigma Aldrich, #D9542) for 1.5 h at room temperature. Imaging was performed on a Zeiss LSM710 confocal microscope, and images were analyzed using ImageJ software.

### 4.9. Semiquantitative Real-Time PCR (sqRT-PCR)

hKC or JEB-KC (2 × 10^5^) were seeded in 6 wells. JEB-KC were either mock-treated (only liposomes) or AON-treated and incubated for 48 h. Cells were harvested in lysis buffer for RNA extraction using the RNeasy Mini Kit (QIAGEN, #74104). cDNA was prepared from 1-µg RNA using the iScript™ cDNA Synthesis Kit (Bio-Rad, #1708891). sqRT-PCR was performed in a Bio-Rad C1000 Touch™ Thermal Cycler CFX96™ Real-Time System using the GoTaq^®^ qPCR Master Mix (Promega, #A600A, madison, WI, USA). Primers were designed to bind at the 3′ end (exon 51/exon53) of *COL17A1*. *GAPDH* was used as the reference gene.

C17_FW: 5′-GGAGCGAGCTGATCAGCTACCTCAC-3′
C17_RV: 5′-GCCATCCCTTGCAGTAGGCCCTG-3′
GAPDH_FW: 5′-GCCAACGTGTCAGTGGTGGA-3′
GAPDH_RV: 5′-CACCACCCTGTTGCTGTAGCC-3′

### 4.10. NGS

We designed an AON-specific AmpliSeq panel to perform NGS analyses on the Ion Torrent Personal Genome Machine (PGM) platform. A setup of a 400-bp customized panel, including a target of 175 bp, was chosen with forward primer 5′-AAACAAAGCCTGACTCATGGCA-3′ and reverse primer 5′-CTCACACTTGCCGATCGACTC-3′ to cover exons 4–9 of the *COL17A1* gene. A mean vertical coverage of about 284 reads was attained. Library and template preparation, as well as sequencing, were performed according to the manufacturer’s protocols (Thermo Fisher Scientific/Life Technologies, Carlsbad, CA, USA). Data analysis was implemented on Integrative Genome Viewer (IGV).

### 4.11. Western Blot Analysis

Protein extracts were generated from ~ 2.5 × 10^5^ cells at a confluency of 80%, dissolved and homogenized in 300-µL cell lysis buffer (0.5-M Tris-HCl, pH 6.8, 20% glycine, 10% SDS, 5% β-mercaptoethanol, 1× protease inhibitor (Roche, #11836153001, Basel, switzerland) and 1x phosphatase inhibitor (Cell Signaling, #5870S, Danvers, MA, USA)). Proteins were separated on a 4–15% bis-tris gel (Invitrogen, Nupage, NP0323) in a MOPS buffer and blotted onto a 0.45-μm nitrocellulose membrane (Amersham Biosciences, Hybond-ECL, Merck, GERPN303D). Ponceau Red staining was performed for the total protein analysis of ChemiDoc (Bio-Rad) images in ImageLab software (Bio-Rad, v5.2.1). Membranes were blocked using 5% milk powder in tris-buffered saline (TBS)/0.1% Tween-20 (Merck, 655205-250ML, Darmstadt, Germany). Primary antibodies were incubated overnight at 4 °C (C17 rabbit monoclonal EPR18614, abcam, #ab184996 and ANXAI mouse monoclonal (EH17a) (Santa Cruz, #sc-12740, Santa Cruz, CA, USA)). Membranes were washed five times using TBS/0.1% Tween-20. Secondary, HRP-labeled antibodies were incubated for 2 h at room temperature (EnVision+ System HRP-labeled polymer anti-rabbit (Dako, #K4003, Santa Clara, CA, USA) and EnVision+ System HRP-labelled polymer α-mouse (Dako, #K4004)). HRP activity was visualized using the Amersham ECL Select Western blot detection reagent (Amersham Biosciences, RPN2235) on a ChemiDoc system (BioRad).

### 4.12. Transfection of keratinocytes with AONs

For AON transfections, lipoplexes at a ratio of 16:1 were generated, as described above. Cells (2 × 10^5^) were seeded per well into a 6-well culture plate and incubated in 4-mL serum-free medium at 37 °C and 5 % CO_2_ in a humidified atmosphere. One hundred microliters of the lipoplex solution were added dropwise, and the cells were incubated at 37 °C for 4 h. Medium was changed, and the cells were incubated for 24–48 h until harvesting.

### 4.13. Conditioning of Cell Culture Supernatants

Cells were cultured without medium change for three to four days in 10-mL serum-free medium (CELLnTEC, #CnT-PR) in T75 flasks until about 90% confluence. Conditioned medium was filtered through a cell strainer, and proteins were precipitated by adding 4 mL of a saturated (100%), ice-cold ammonium sulfate (AS) solution dropwise to an 8-mL culture medium. The addition of AS was conducted over a period of 5–10 min. After adding AS, the solution was incubated at 4 °C overnight with overhead rotation. The next day, the solution was centrifuged at 5000× *g* for 30 min at 4 °C. The supernatant was aspirated, and the pellet was resuspended in 30–50-µL 8M urea in TBS. Since no loading control was available for the conditioned medium, Ponceau staining was used to determine the equal protein loading.

### 4.14. 3D Organotypic Skin Equivalents

For the construction of skin equivalents (SEs), 1-mL Cnt Prime medium was mixed with 500-µL fibrinogen (25 mg/mL; Sigma, #F4883), 100-µL aprotinin (ready to use stock; Sigma, #A6279-5ML) and 100-µL thrombin (~1 NIH unit; Sigma, #T8885) and cast into a standard 6-well culture plate. After polymerization for 1h at 37 °C, the fibrinogen matrix was coated with LN-511 (2.5 µg/cm^2^, BioLamina, Sundbyberg, Sweden) overnight at 4 °C. The next day, 10^6^ keratinocytes were seeded onto the gel matrix and incubated until confluency in Green’s Medium + 100-µg/mL ascorbic acid. After 2 days, the culture inserts were placed in deep well 6-well plates (CORNING, #355467, Corning, NY, USA) and cultured at the air–liquid interface for one week using Green’s Medium with 100-µg/mL ascorbic acid. Medium was changed to a differentiation medium after 7 days: DMEM + Ham’s F12 3:1, 1% FCII, 0.16% BSA, 4-mM L-glutamine, 0.1-mM ethanolamine, 1-mM Na-pyruvate, 50-µg/mL hydrocortisone, 5-µg/mL insulin, 0.14-mM adenine, 1.37-pg/mL triiodothyronine, 10-mM L-serine, 10-µM L-carnitine, 1% penicillin/streptomycin and, at every medium change, 100-µg/mL ascorbic acid were added freshly. JEB-KC patient skin equivalents were treated once with lipoplexes of a 16:1 ratio. After one week in differentiation medium, SEs were harvested and OCT (Scigen, Tissue-Plus, Paramount, CA, USA) embedded.

### 4.15. Liposomal Delivery of AONs into Human Skin Explants

The skin samples were obtained from patients undergoing dermatological surgery upon written, informed consent. The fatty layer was removed with a scalpel, and the skin was cut into sections of approximately 3 × 3 cm and washed in 1× antibiotic/antimycotic and twice in PBS. Skin samples where either tape-stripped 30 times with a highly adhesive duct tape or left untreated. The skin samples were placed in a 10-cm petri dish on a piece of gauze, soaked in DMEM and the bottom of the dish was filled with DMEM to avoid dehydration of the skin during the incubation period. Under gentle but constant vortexing, 160-µg DDC642 were mixed with 10-µg Cy5-AON; the solution was vortexed for a further 15 s and then incubated at room temperature for 10 min. The lipoplex solution was administered as a single drop in the middle of each skin piece, the cover was placed on the dish and the samples were incubated for 24 h at room temperature. The next day, the remnants of the lipoplex solution were removed from the skin with a PBS-soaked swab, and 6-mm punch biopsies were taken to generate cryosections for fluorescence microscopy.

## 5. Conclusions

In conclusion, we showed successful exon skipping within the intracellular domain of C17 and, consequently, the re-expression and correct deposition of the protein in a 3D setting. Given the first marketing approvals for AONs in general and first clinical trials using AONs also in an EB context renders the above-described molecules also as potential candidates to be taken on in the clinical setting, which would contribute a new JEB treatment option and also demonstrate the feasibility of the development therapies for ultra-rare EB types.

## Data Availability

The data that support the findings of this study are available upon request.

## Supplementary Materials

The following are available online at https://www.mdpi.com/1422-0067/22/7/3326/s1.
