# Supplementary material for: Personalized Development of Antisense Oligonucleotides for Exon Skipping Restores Type XVII Collagen Expression in Junctional Epidermolysis Bullosa
Source: Int J Mol Sci. 2021 Mar 24;22(7):3326. doi: 10.3390/ijms22073326 (PMC8036626; doi:10.3390/ijms22073326)
Supplement: Supplementary file 1 [file ijms-22-03326-s001.pdf]

Supplementary Information - Article

Personalized development of antisense-oligonucleotides for exon skipping restores type XVII collagen function and expression in junctional epidermolysis bullosa

Michael Ablinger[1*], Thomas Lettner[1*], Nicole Friedl[1], Hannah Potocki[1], Theresa Palmetzhofer[1], Ulrich Koller[1], Julia Illmer[1], Bernadette Liemberger[1], Stefan Hainzl[1], Alfred Klausegger[1], Manuela Reisenberger[1,3], Jo Lambert[2], Mireille Van Gele[2], Eline Desmet[2], Els Van Maelsaeke[2], Monika Wimmer[1], Roland Zauner[1], Johann W. Bauer[1,3], Verena Wally[1].

[1] Research Program for Molecular Therapy of Genodermatoses, EB House Austria, Department of Dermatology and Allergology, University Hospital of the Paracelsus Medical University, 5020 Salzburg, Austria.

[2] Department of Dermatology, Ghent University Hospital, 9000 Ghent, Belgium.

[3] Department of Dermatology and Allergology, University Hospital of the Paracelsus Medical University, 5020 Salzburg, Austria.

* Equally contributing authors.

Correspondence: v.wally@salk.at; Tel.: +43 5 7255 82400

Supplementary Figure S1. Characterization of JEB-KC.

Supplementary Figure S2: Scheme of AON binding sites and splice pattern in JEB-KC.

Supplementary Figure S3. Generation and characterization of lipoplexes.

Supplementary Figure S4. DDC642 delivery of AONs to human skin.

Supplementary Figure S5. Control treatments of skin specimens.

References

Supplementary Figure S1. Characterization of JEB-KC. A) A splice site mutation at the intron 6, exon 7 junction leads to skipping of the first 16 nucleotides of exon 7 during pre-mRNA processing, leading to an out-of-frame transcript that undergoes NMD. **B)** sqRT-PCR confirmed skipping of 16 nts, which **C)** results in overall reduced *COL17A1* expression levels (unpaired, two-sided Student's t-test, $p < 0.0001$). **D)** Western blot analysis confirmed reduced expression of type-XVII collagen in JEB-KCs. ANXAI was used as loading control. ($n > 3$).

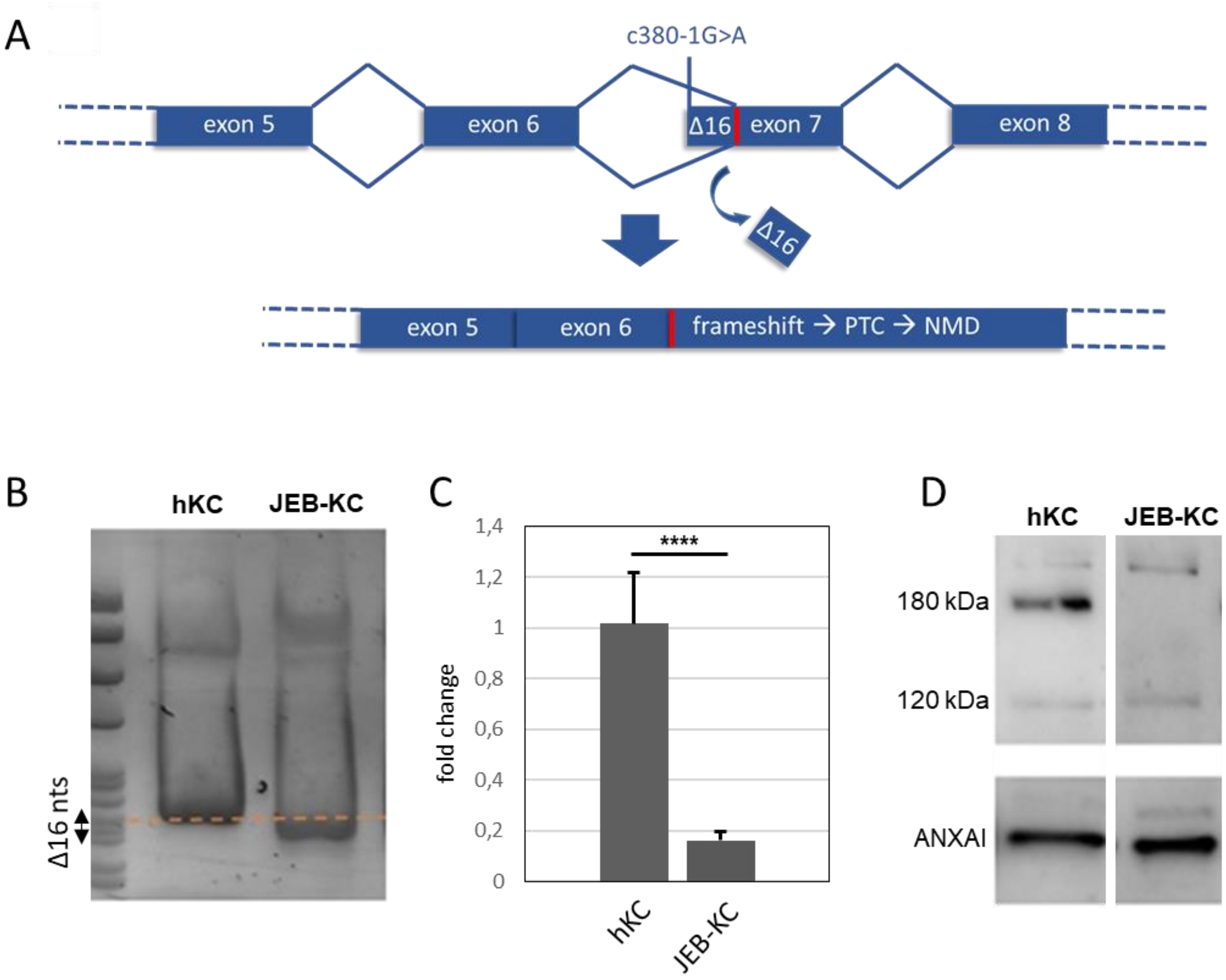

Supplementary Figure S2. Scheme of AON binding sites and splice patterns in JEB-KC. A) ESEfinder 3.0 [1] analyses displaying the *COL17A1* JEB-KC and **(B)** wild-type intron 6 / exon 7 junction sequence and binding sites of AONs. Color code indicates the best putative binding sites for SRSF splicing-associated proteins. The mutated nucleotide c.380-1G>A is framed in red.

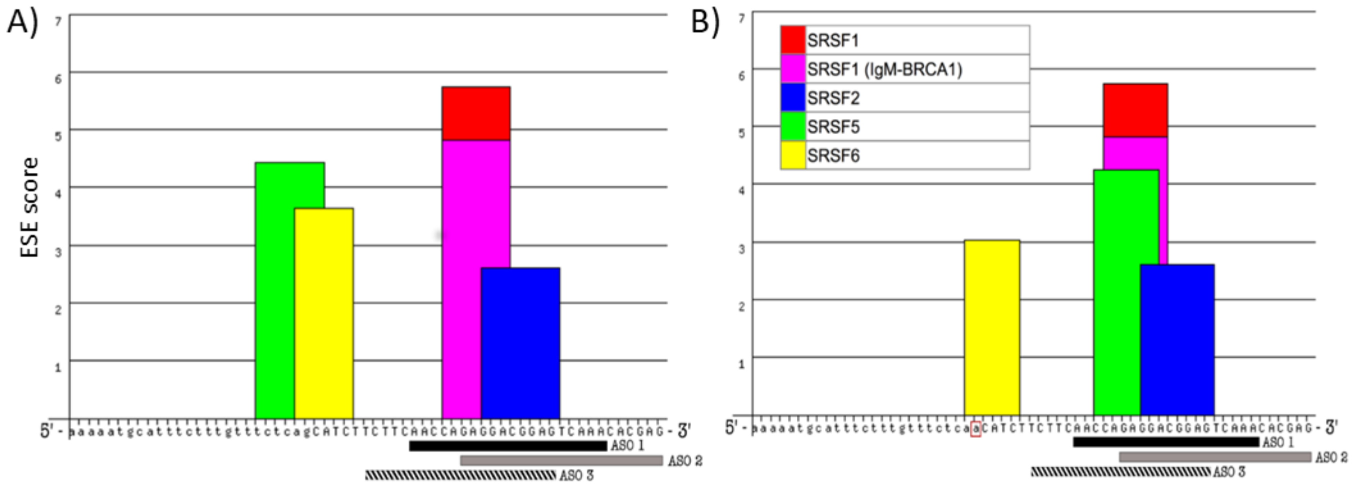

Supplementary Figure S3. Generation and characterization of lipoplexes. A) AONs efficiently form complexes with DDC642 liposomes at the ratios 16:1 and 32:1 (liposome:AON), as indicated by the absence of free AONs. **B)** In an MTT assay, no significant impact of liposomes on keratinocyte viability was evident. (n = 4) **C)** Treatment of JEB-KCs with Cy5-AON lipoplexes indicated transfection efficiencies of up to 90%, as determined by cell-based fluorescence imaging and automated calculating Cy5-positive cells over DAPI. Cy5-labelled ASOs localized to the nucleus. Scale bars: 50 µm.

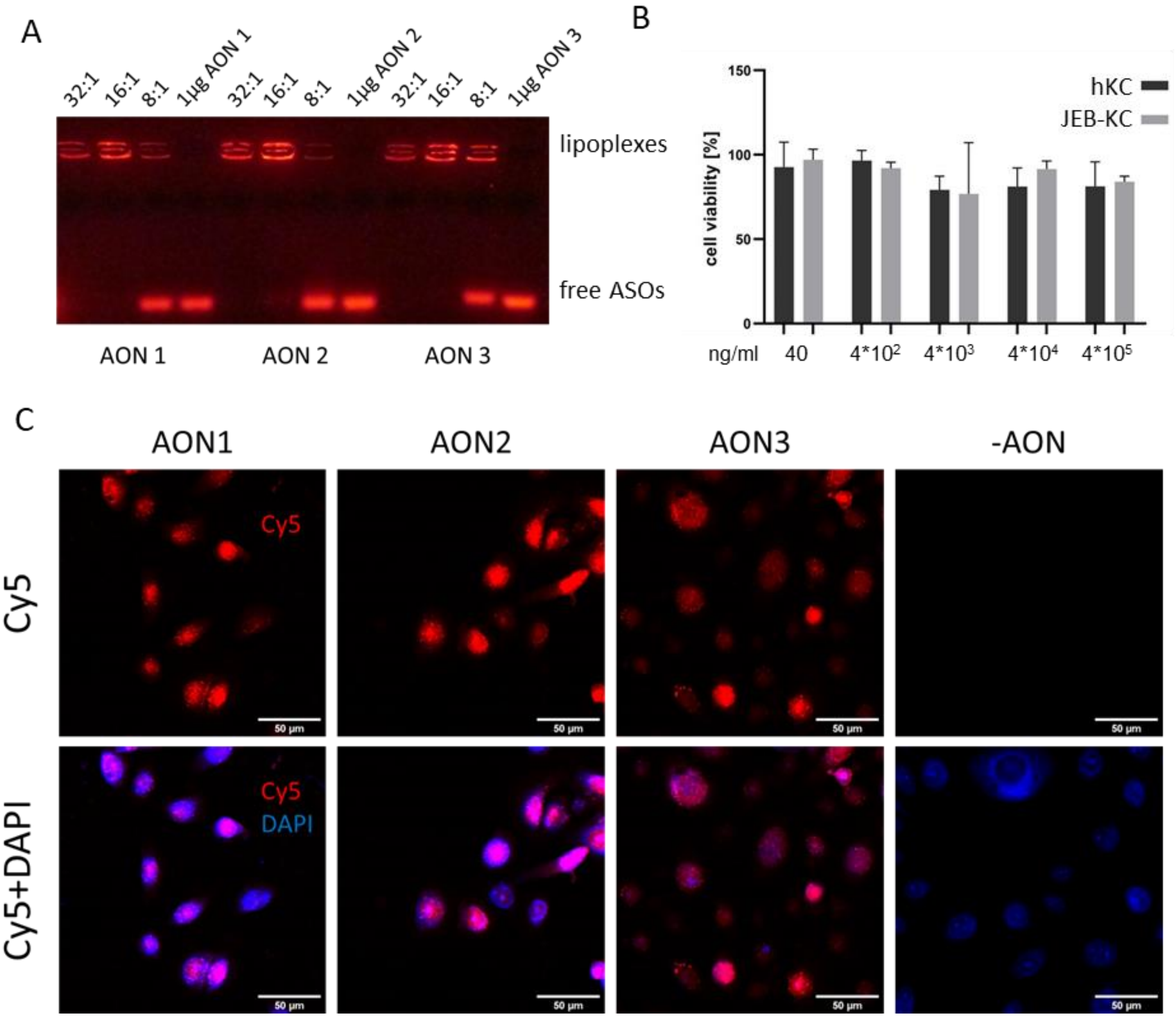

Supplementary Figure S4. DDC642 delivery of AONs to human skin. In order to test DDC642-AON delivery into human epidermis, Cy5-AON lipoplexes were administered onto skin biopsies of a healthy donor **(A)**. In addition, intra-dermal injection of lipoplexes **(B),** and administration onto tape stripped skin **(C)** was tested. **(D)** Liposomes without Cy5-AONs were used as negative control on tape stripped skin. Dotted lines indicate the junction between epidermis and dermis. Magnification with **(E-H)** or without DAPI **(I-L)** shows different localization of Cy5-signal according to pre-treatment of the skin. While in untreated skin there is a strong staining of the stratum corneum and hardly any staining of the dermis **(A,E,I)**, there is a homogenous distribution of the Cy5 signal in the whole section upon intradermal injection, which might bare a safety risk of systemic distribution and associated immune reactions **(B,F,J)**. Upon tape stripping **(C,G,K)**, predominantly the epidermis shows Cy5 fluorescence. **(M-O)** Immunohistochemistry of mechanically untreated sections (M), as well as upon intradermal injection **(N)** and tape stripping **(O)**. Scale bars A-D: 50 µm. Scale bars E-L: 20 µm. Scale bars M-O: 100 µm.

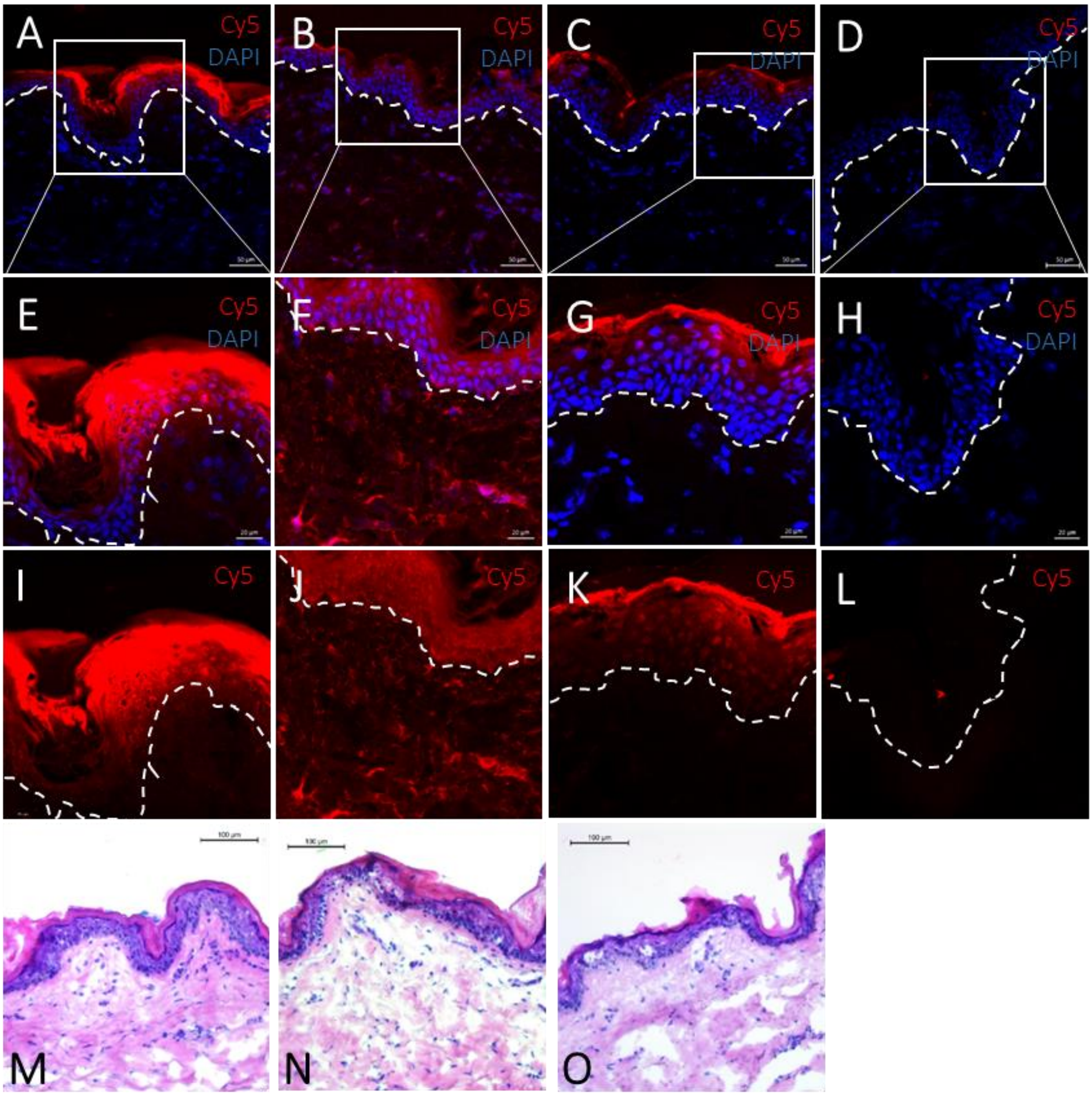

Supplementary Figure S5. Control treatments of skin specimens. As a control for analyzing the delivery potential of DDC642 liposomes, we applied naked AONs onto skin explants. Red fluorescence remained predominantly restricted to the stratum corneum when AONs were applied onto (**A)** tape stripped skin, or on (**B)** skin with no pre-treatment. **C)** Upon intradermal injection, AONs remained at the site of injection. Scale bars: 20 µm. **D-F)** Respective H&E stainings are shown. Scale bars: 100 µm.

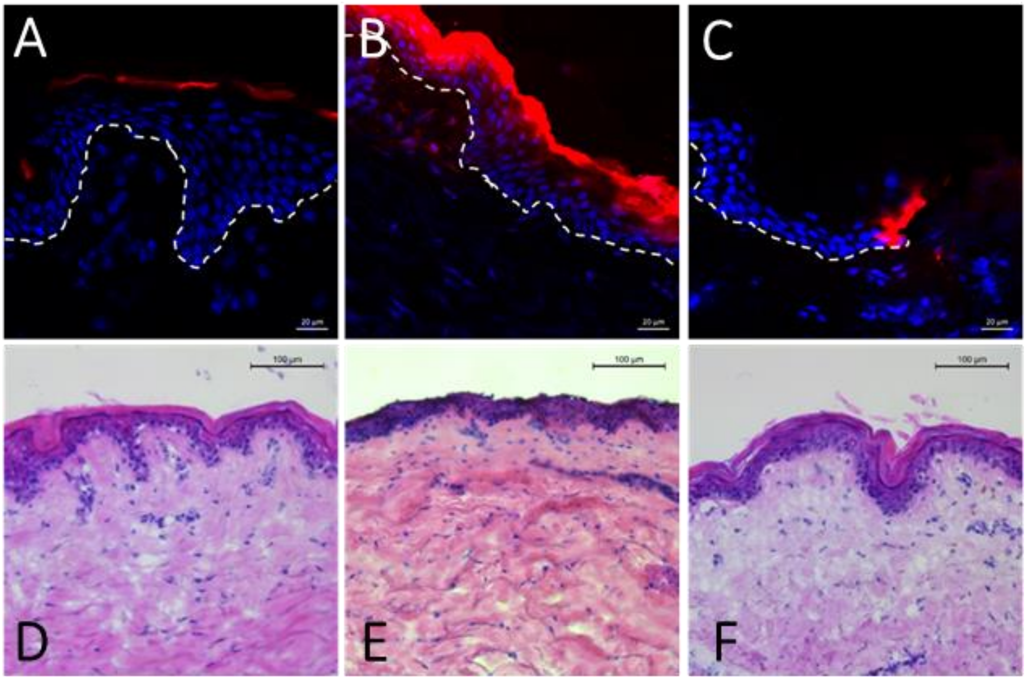

References:

1 Smith PJ, Zhang C, Wang J, Chew SL, Zhang MQ, Krainer AR. An increased specificity score matrix for the prediction of SF2/ASF-specific exonic splicing enhancers. Hum Mol Genet. 2006 Aug 15;15(16):2490-508. doi: 10.1093/hmg/ddl171. Epub 2006 Jul 6. PMID: 16825284.
